# Face-to-face interference in typical and atypical development

**DOI:** 10.1111/j.1467-7687.2011.01125.x

**Published:** 2012-03

**Authors:** Deborah M Riby, Gwyneth Doherty-Sneddon, Lisa Whittle

**Affiliations:** 1 School of Psychology, Newcastle UniversityUK; 2 School of Life Sciences, Northumbria UniversityUK; 3 Department of Psychology, Stirling UniversityUK

## Abstract

Visual communication cues facilitate interpersonal communication. It is important that we look at faces to retrieve and subsequently process such cues. It is also important that we sometimes look away from faces as they increase cognitive load that may interfere with online processing. Indeed, when typically developing individuals hold face gaze it interferes with task completion. In this novel study we quantify face interference for the first time in Williams syndrome (WS) and Autism Spectrum Disorder (ASD). These disorders of development impact on cognition and social attention, but how do faces interfere with cognitive processing? Individuals developing typically as well as those with ASD (n = 19) and WS (n = 16) were recorded during a question and answer session that involved mathematics questions. In phase 1 gaze behaviour was not manipulated, but in phase 2 participants were required to maintain eye contact with the experimenter at all times. Looking at faces decreased task accuracy for individuals who were developing typically. Critically, the same pattern was seen in WS and ASD, whereby task performance decreased when participants were required to hold face gaze. The results show that looking at faces interferes with task performance in all groups. This finding requires the caveat that individuals with WS and ASD found it harder than individuals who were developing typically to maintain eye contact throughout the interaction. Individuals with ASD struggled to hold eye contact at all points of the interaction while those with WS found it especially difficult when thinking.

## Introduction

Faces provide visual communication signals that need to be interpreted to facilitate human interactions (Clark & Brennan, 1991). Such signals may take the form of a shift of eye gaze to indicate turn taking or a change of emotional expression to indicate thoughts or feelings and these can occur alongside other non-verbal gestures. During social communication we need to look at our interlocutor to detect and subsequently decipher this range of sophisticated inter-personal signals. There is no doubt that if these signals are missed there will be a negative impact on the flow of the interaction. Older children and adults can communicate effectively in audio-only interactions, such as talking on the telephone. However, in order to do so they must adapt their interactive style. For example, when visual communication cues are not available more words and turns are typically used to reach the same communicative outcome. Doherty-Sneddon and Kent (1996) report that young children (6-year-olds) communicate significantly less effectively in unseen interactions perhaps because they are unable to use cues such as hand gestures and eye gaze. There is, therefore, evidence that visual communication cues provide important information in human communication.

Throughout typical development, looking at faces is important to learn an array of social signals and visual communication cues. However, looking away from faces at key points of an interaction is also critical. Holding mutual face gaze carries a cognitive load because of the rich information that is provided, and in certain circumstances we need to reduce this cognitive load and free resources to complete other tasks (e.g. Doherty-Sneddon, Bruce, Bonner, Longbotham & Doyle, 2002). A shift of eye gaze away from the face of another person, usually an interlocutor, is termed ‘gaze aversion’ (Doherty-Sneddon *et al*., 2002; Glenberg, Schroeder & Robertson, 1998). Typically, we spontaneously look away from the face of a companion during cognitively demanding activity by engaging in overt gaze aversion (GA). Individuals who are developing typically tend not to employ GA when they are listening to a speaker, as visual cues can facilitate communication (e.g. lip movements facilitate speech perception; McGurk & MacDonald, 1976). However, at other points of an interaction (especially when thinking, but also when speaking; Ehrlichman, 1981; Kendon, 1967) it may be more beneficial to avert gaze than to subject ourselves to the additional load associated with holding face gaze. It has been proposed that GA reflects the requirement to concentrate on drawing information from memory and / or engaging in on-line cognitive processing, such as speech-planning or computation. Averting gaze may be as important functionally for processing information as holding mutual gaze is for detecting visual signals that can benefit communication.

What happens to cognitive processing if we do not avert gaze and attempt to hold mutual eye contact whilst also processing information? As adults, if we are encouraged to look continuously at a listener while we are speaking, our speech becomes disjointed, less fluent and may finally become derailed (Beattie, 1981). Earlier in development, research with typically developing children has also suggested that face-to-face contact can interfere with cognitive processing (Doherty-Sneddon, Bonner & Bruce, 2001). ‘Forcing’ a child to look at a person’s face while they listen to descriptions of abstract shapes interferes with their ability to understand the description given to them (accuracy decreases and reaction times increase). Children perform the same task much better if they are able to look away from a face when they are thinking (e.g. looking at the floor; Doherty-Sneddon *et al*., 2001, Experiment 1). Children also show poorer visuo-spatial memory abilities when they are required to look at faces compared to the floor or a moving abstract pattern (Doherty-Sneddon *et al*., 2001; Experiment 2a). The difference between the face and abstract pattern condition emphasizes the critical role of the human face in any interference, as opposed to the presence of any visually complex stimuli and as opposed to the mere presence of performing a ‘dual task’. Having said this, Glenberg *et al*. (1998) report gaze aversion away from questions written on computer screens during ‘thinking time’. It is, therefore, likely that faces are only one source of distraction in the visual environment (albeit a particularly salient one, e.g. Doherty-Sneddon *et al*., 2001). Faces are rich in socially relevant information and thus interfere with cognitive processing capacity. Some would go as far as to say that averting gaze benefits cognitive performance not only due to the disengagement of attention from a complex stimulus, but also because of the interruption of demanding social interaction processes involved in face-to-face communication (e.g. Markson & Paterson, 2009). Research emphasizes the role of visual communication signals in social interactions throughout typical development. Furthermore, such evidence has led some researchers to advocate teaching children gaze aversion strategies to enhance learning (Doherty-Sneddon & Phelps, 2005; Phelps, Doherty-Sneddon & Warnock, 2006).

It is important to note that a related position sees GA as a ‘by-product’ of cognitive processing (Previc, Declerck & de Brabander, 2005; Previc & Murphy, 1997). Eleven-year-old children and adults have been found to show a high prevalence of upward eye movements in response to a variety of question types. Previc and colleagues propose that these upward lateral eye movements (LEM) reflect engagement in demanding cognitive activity and share common underlying neural circuitry with higher cognition (e.g. abstract thought). Accordingly, Previc and colleagues argue that there is a link between upward LEMs and thought because both make use of saccadic neural machinery in the lateral prefrontal cortex and other oculomotor association cortical regions, regions which are of relevance to higher human thought because of their orientation to distant space (Pierrot-Deseilligny, Muri, Ploner, Gaymard & Rivaud-Pechoux, 2003) and association with working memory (Kane & Engle, 2002). This by-product model clearly contrasts with the ‘cognitive load’ hypothesis (Glenberg *et al*., 1998; Doherty-Sneddon *et al*., 2002). Of course these divergent approaches may indeed be consonant with one another: GA may reflect the nature of the cognitive activity that it benefits.

How about *atypical* development and the interaction between attention to faces and cognitive processing? There are two neuro-developmental disorders that are key candidates for this exploration due to diverse and atypical patterns of spontaneous attention to faces; namely, Williams syndrome (WS) and Autism Spectrum Disorder (ASD). These disorders of development are associated with atypical patterns of gaze behaviour, atypicalities of social functioning and intellectual impairment. We take each disorder in turn to consider how they may inform theories of typical gaze behaviour and cognition, but also how insights into the impact of mutual face gaze on cognition may inform understanding of behaviours associated with these specific disorders.

The autism spectrum comprises a variety of disorders characterized by marked deficits in communication and social interactions, as well as restricted interests and repetitive behaviours (APA, 2000). Communication impairments may include a delay or lack of development of spoken language while social impairments are likely to encompass the poor modulation of eye contact, deficits interpreting facial affect, and a lack or atypicality of gesture during social interactions. Many individuals with an ASD fail to develop peer relations as a consequence of poor social and emotional reciprocity. Atypicalities of attention to social information are particularly evident. For example, reduced attention to faces has been widely reported in ASD, especially when there is competition for attentional resources from non-social information (Klin, Jones, Schultz, Volkmar & Cohen, 2002; Speer, Cook, McMahon & Clark, 2007; Riby & Hancock, 2008, 2009; Kikuchi, Senju, Tojo, Osanai & Hasegawa, 2009; but see also Fletcher-Watson, Leekam, Turner & Moxon, 2006; Freeth, Ropar, Chapman & Mitchell, 2010). These atypicalities occur even when attending to images on a computer screen when there is no opportunity for holding mutual gaze with another individual engaged in a real interaction. Therefore, the realism of an interaction may further exacerbate any atypicality of face gaze associated with ASD. This is no more important than during real-life interactions when subtle and spontaneous visual signals may be critical to smooth running interactions. It is widely recognized that individuals with an ASD exhibit atypical eye contact patterns during real-life social interactions that may be evident from a young age (Dawson, Toth, Abbott, Osterling, Munson, Estes & Liaw, 2004). Difficulties modulating gaze during a social interaction are reported (Willemsen-Swinkels, Buitelaar, Weijnen & Engeland, 1998) and failure to attend to socially relevant information in a typical manner will impact upon the development of social expertise (Klin, Jones, Schultz & Volkmar, 2003).

Theoretical insights into the mechanisms driving reduced or atypical face gaze in ASD have been postulated (for a full discussion refer to Senju & Johnson, 2009). For example, while some researchers support a ‘hyperarousal’ viewpoint, whereby faces increase arousal which is uncomfortable and thus faces are highly aversive (Dalton, Nacewicz, Johnstone, Schaefer, Gernsbacher, Goldsmith, Alexander & Davidson, 2005), others suggest that individuals with ASD are indifferent to faces and do not show a ‘typical’ appreciation for socially oriented information (Dawson, Webb & McPartland, 2005). Recent research using human interactions has suggested a lack of support for the aversive nature of faces as individuals are able to make eye contact at a typical level during a social interaction (Doherty-Sneddon, Riby & Whittle, in press). However, the same study reports increased and atypical gaze aversion (looking away from a face) when individuals with ASD are listening to information from the experimenter, suggesting a lack of awareness of the significance of receiving visual cues or that individuals with ASD need to start computing information earlier in the interaction due to working memory and executive functioning demands. If individuals with ASD showed hyper-arousal to faces driving inattention towards them, we would expect elevated levels of GA across the whole interaction. To date, research has not quantified the association between cognitive performance and eye contact in this population. The current work attempts to quantify this difficulty in relation to typically developing individuals as well as another neuro-developmental disorder.

Compared to ASD, Williams syndrome (WS) is a relatively rare neuro-developmental disorder with an estimated prevalence of between 1:7,500 (Strømme, Bjørnstad & Ramstad, 2002) and 1:20,000 (Morris & Mervis, 1999). The disorder is caused by a sporadic 1.5 MB deletion that includes about 24–28 genes on chromosome 7 (7q11.23; Donnai & Karmiloff-Smith, 2000). WS is associated with mild to moderate intellectual impairment (Searcy, Lincoln, Rose, Klima, Bavar & Korenberg, 2004) that occurs in parallel with unique cognitive and socio-behavioural phenotypes. While the uneven cognitive phenotype has captured the attention of cognitive scientists (for a review see Martens, Wilson & Reutens, 2008), the social characteristics associated with WS have spurred comparisons and contrasts with ASD. Importantly, social behaviour is atypical in both WS and ASD although the nature of the atypicalities is very different (for a discussion see Brock, Einav & Riby, 2008). WS has been linked to reports of outgoing hypersocial behaviour (Jones, Bellugi, Lai, Chiles, Reilly, Lincoln & Adolphs, 2000; Doyle, Bellugi, Korenberg & Graham, 2004; Frigerio, Burt, Gagliardi, Cioffi, Martelli, Perrett & Borgatti, 2006), indiscriminate approach to strangers and, important for the current study, atypically prolonged attention to faces (Mervis, Morris, Klein-Tasman, Bertrand, Kwitny, Appelbaum & Rice, 2003; Riby & Hancock, 2008, 2009). Although faces may not capture attention particularly fast in WS, once they have grabbed attention, disengagement may be more difficult (Riby & Hancock, 2009; Riby, Jones, Robinson, Langton, Bruce & Riby, 2011). Having said this, during a face-to-face interaction individuals with WS are able to modulate their gaze and they display a typical pattern of increased gaze aversion when thinking about cognitively demanding information (Doherty-Sneddon *et al*., in press). Research to date has not explored or quantified the impact of maintaining face gaze on cognition in this group.

In this study we question whether enforcing face gaze interferes with cognitive capacity in the neuro-developmental disorders WS and ASD and whether this occurs in a manner similar to that seen in typical development (children, Doherty-Sneddon *et al*., 2001; adults, Markson & Paterson, 2009). Participants are encouraged to maintain eye contact with an interlocutor when they are listening to, thinking about and answering questions. In typically developing individuals GA is most frequent during thinking and thus serves to free up cognitive resources to complete the task at hand (Doherty-Sneddon *et al*., 2002). This pattern has also recently been reported in WS and ASD (Doherty-Sneddon *et al*., in press). The natural next step with these populations is to consider the interference caused by holding face gaze, compared to the interference seen in typical development. We hypothesize that in ASD sustained face gaze will be difficult and will cause significant detriments to cognitive performance (see Attwood, 1998) when compared to the effects seen in typical development. We propose that in WS sustained face gaze will impact upon cognitive performance, but that it may not be so difficult for this group to hold face gaze, compared to the difficulty seen in typical development, given reports of atypically prolonged attention to faces on computer screens (Riby & Hancock, 2008) and during everyday social interactions (Mervis *et al*., 2003). Finally, it is anticipated that typically developing participants will replicate the pattern reported in previous research, whereby holding face gaze reduces task accuracy.

## Method

### Participants

Nineteen participants who had previously been diagnosed with an Autism Spectrum Disorder (ASD; 17 males) ranged from 12 to 17 years of age (mean 14 years 10 months). Participants were recruited from a special education unit of a mainstream secondary school, a school for pupils with additional educational needs, and Dasl^n^e, a database for individuals with autism spectrum disorders living in the north-east of England (http://www.daslne.org). Parents confirmed that their child had previously been diagnosed with an Autism Spectrum Disorder by a clinician according to the DSM-IV criteria (APA (American Psychiatric Association), 2000). Parents were also asked to complete the Social Communication Questionnaire (SCQ-Lifetime; Rutter, Bailey & Lord, 2003), a 40-item parent-report screening measure probing autistic symptomatology. The SCQ has gained support for its use in both research and clinical settings. Parents of 12 individuals in the ASD group successfully completed the questionnaire and SCQ scores ranged from 17 to 30 (group mean 24; clinical cut-off is 15; note that all individuals scored above the cut-off for an ASD). Parents of the remaining six individuals with ASD confirmed that their child had a diagnosis of ASD, and this was further supported by teachers working with the individual. However, they provided incomplete SCQ data. On the British Picture Vocabulary Scale II (BPVS II; Dunn, Dunn, Whetton & Burley, 1997) the ASD group scored between 59 and 138 (raw scores) and had a mean verbal mental age of 10 years and 7 months (mean raw score 101, standard deviation 22.14).

Sixteen participants who had previously been diagnosed with WS (11 males) ranged from 9 to 37 years (mean 22 years 6 months) and were recruited via the Williams Syndrome Foundation. All participants had previously been diagnosed phenotypically and all had previously had their diagnosis confirmed genetically with positive fluorescent in situ hybridization (FISH) testing to detect the deletion of one copy of the ELN gene in the long arm of chromosome 7. On the BPVS II (Dunn *et al*., 1997) the WS group scored between 71 and 132 (raw scores) and had a mean verbal mental age of 10 years and 2 months (mean raw score 99, standard deviation 16.37).

Each individual with ASD or WS was matched to a typically developing participant on the basis of verbal ability using raw scores on the BPVS II (Dunn *et al*., 1997). All typically developing participants complied with the inclusion criteria by scoring within the normal behaviour range on the Strengths & Difficulties Questionnaire (Goodman, 2001) completed by teachers (all scoring below a total of 11). The typically developing participants that were matched to the ASD group on the basis of BPVS II had raw scores between 58 and 136 (mean 101, standard deviation 21.16). Chronologically, this group was aged between 5 and 15 years (mean 10 years 10 months; 10 males). The typically developing participants that were matched to the WS group on the basis of BPVS II had scores between 74 and 128 (mean 99, standard deviation 16.37). Chronologically, this group was aged between 7 and 15 years (mean 10 years 6 months; 8 males). The neuro-developmental disorder groups and their typically developing matches did not differ on BPVS II raw scores (ASD-TD *p* = .98; WS-TD *p* = .96). The ASD and WS groups were not matched to each other.

Participants in all groups had normal or corrected-to-normal vision. Parent consent, and where appropriate participant assent, was obtained prior to participation. Participants were tested in a quiet environment at their home or in their school.

### Materials and design

Gaze behaviour was recorded during a question and answer session. The experimenter and the participant sat opposite each other (approximately 1 to 1.5 metres apart, facing each other). A video recorder was set up behind the experimenter to monitor the gaze behaviour of the participant throughout the session. The gaze data would subsequently be analysed to confirm adherence to instructions and explore gaze behaviour.

The testing session was split into two phases (overall length approximately 15–20 minutes). In both phases the experimenter asked the participant a series of mental arithmetic questions. The participant was required to listen to each question (listening phase), work out their answer (thinking phase), and then provide their response to the experimenter (speaking phase). The listening phase ended when the experimenter stopped speaking. At this point the thinking phase began and ended when the participant began speaking their response. The questions were rated as moderately difficult for each participant, with parents and teachers modifying the questions to the child’s needs prior to the session. Parents of individuals with WS, and teachers of the ASD and TD groups, thoroughly discussed the child’s mathematics ability with the experimenter prior to the study (e.g. answering and discussing, can the child add numbers up to 10? Can the child add numbers up to 20? Can the child compute subtractions?). Question difficulty was thus set according to individual abilities (this is important when including populations such as WS where numeracy may be problematic; see Appendix for example questions). Importantly, all participants were engaged in answering the questions and would be able to achieve equivalent (moderate) levels of success. It is also worth noting that questions rated as ‘moderately difficulty’ were those that were found to be most amenable to improvements when typically developing children were trained to increase their gaze aversion in previous research (see Phelps *et al*., 2006). Six mathematics questions were asked during each phase of the experiment. Accuracy of responses (% correct) was recorded by the experimenter as the primary dependent variable.

The only difference between phases 1 and 2 was the gaze instruction given to the participant. All participants completed phase 1 prior to phase 2.^1^ During phase 1 there was no constraint on the participant’s gaze direction and no instruction was given relating to eye gaze (‘gaze at will’ condition). In phase 2, participants were specifically asked (and subsequently prompted) to hold eye contact with the experimenter during the interaction (‘eye contact’ condition). As previously noted, gaze behaviour was recorded and subsequently coded on a frame-by-frame basis for (i) gaze to the experimenter’s face and (ii) gaze away from the experimenter’s face. The length and proportion of gaze aversion (away from the face) during each phase of the interaction formed the dependent variable of secondary interest to the study (being used to confirm adherence to experimental demands).

### Procedure

Participants were told they would be asked some mathematics questions and they were given the following instructions: (i) take as much time as you need to answer each question, (ii) ask if you need a question repeated, and (iii) you will not be given feedback between questions about whether your answer is correct or incorrect. The experimenter ensured that the participant understood the instructions, had an opportunity to ask any questions and had the chance to have a practice.

In both phases, the experimenter looked at the participant at the beginning of each question and maintained eye contact for as long as the participant needed to be able to provide an answer to the question. In phase 2, the participant was told to maintain eye contact at all times (when listening, thinking, and answering the question). A verbal prompt was used before each question was asked (‘keep looking at me’). If the participant averted their gaze in phase 2, a physical prompt (researcher making a hand motion to the eyes) was given and this was followed up with a verbal prompt if needed (‘keep looking at me’). Participants were thanked for their participation at the end of the session.^2^

## Results

We analyse the data from each neuro-developmental disorder group compared to their matched typical comparison group. Our primary outcome measure is the change in accuracy (percentage correct) when asked to maintain eye contact (versus gazing at will). Subsequent analyses explore the pattern of gaze during the interaction to assess adherence to task instructions.

### Task accuracy

#### Autism Spectrum Disorder

The data were subjected to a 2 × 2 analysis of variance (ANOVA) with the repeated factor Gaze Direction (At Will, Eye Contact) and the independent factor Group (ASD, TD). The main effect of Gaze Direction was significant, *F*(1, 36) = 19.02, *p* < .001, as accuracy was significantly reduced in the eye contact condition (overall mean gaze at will, 66%, eye contact 54%). The main effect of Group was not significant (*p* = .81; ASD = 60%, TD = 61%). The interaction between gaze direction and group was not significant (*p* = .45), indicating that accuracy was equally affected by direct eye contact in the ASD and TD groups (see Table 1). Indeed, while the ASD group showed a mean decrease in accuracy of 14% (standard deviation 13.5%) across conditions, individuals who were developing typically showed a mean decrease in accuracy of 10% (standard deviation 18.8%).

**Table 1:** Task accuracy (% correct) as a function of gaze direction and group (standard deviation shown in parentheses)

	Group	

	ASD	TD matches
Gaze – At Will	67 (*12*)	66 (*17*)
Gaze – Eye Contact	53 (*16*)	56 (*28*)
	WS	TD matches
		
Gaze – At Will	69 (*17*)	68 (*20*)
Gaze – Eye Contact	46 (*22*)	52 (*28*)

#### Williams syndrome

The data were subjected to a 2 × 2 ANOVA with the repeated factor Gaze Direction (At Will, Eye Contact) and the independent factor of Group (WS, TD). The main effect of Gaze Direction was significant, *F*(1, 30) = 21.39, *p* < .001, as accuracy was significantly reduced in the eye contact condition (overall mean gaze at will 68%, eye contact 49%). The main effect of Group was not significant (*p* = .69; WS = 57%, TD = 60%). The interaction between gaze direction and group was not significant (*p* = .40), indicating that accuracy was equally affected by direct eye contact in the WS and TD groups (see Table 1). The WS group showed a mean decrease in accuracy of 23% (standard deviation 29%) while the TD group showed a mean decrease of 16% (standard deviation 16.8%).^3^

### Gaze behaviour

#### Autism Spectrum Disorder

A three-way mixed ANOVA was conducted using the gaze aversion data (the percentage of time averting gaze away from the face of the experimenter) with factors Group (ASD, TD), Gaze Direction (At Will, Eye Contact), and Phase of Interaction (listening, thinking, speaking). There was a significant main effect of Group, *F*(1, 36) = 7.81, *p* < .01, as overall individuals with ASD (mean 34%) used more gaze aversion than those developing typically (mean 23%). There was a significant main effect of Gaze Direction, *F*(1, 36) = 163.98, *p* < .001, with more averted gaze in the At Will condition (mean 47%) than the Eye Contact condition (mean 10%). The main effect of Phase of Interaction was also significant, *F*(2, 72) = 85.69, *p* < .001. Post-hoc *t*-tests showed that overall there was less gaze aversion in the listening phase (mean 14%) than the speaking phase (mean 24%; *t*(37) = 3.62, *p* < .01), which in turn showed less gaze aversion than the thinking phase (mean 48%; *t*(37) = 7.72, *p* < .001).

**Figure 1:**
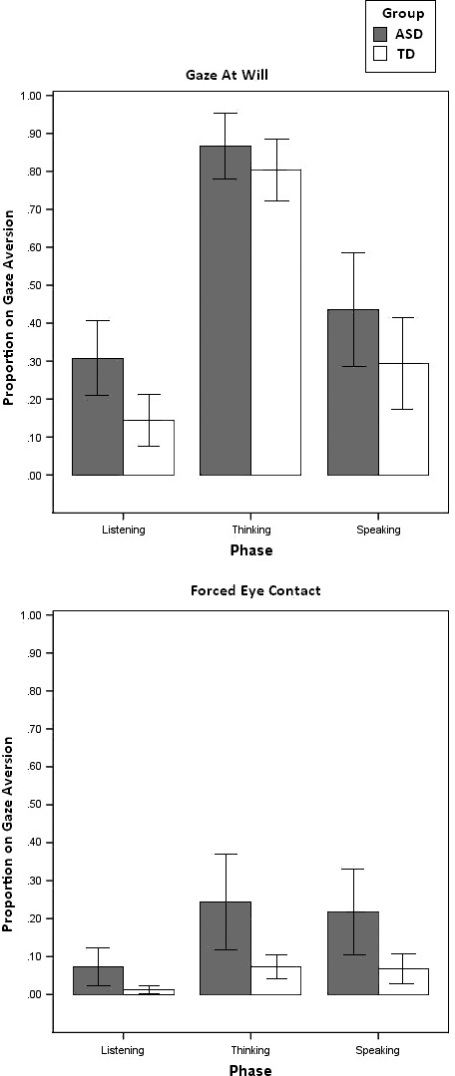
Gaze aversion rates as a function of interaction phase and group for each gaze condition (ASD versus TD). Error bars represent standard error of the mean.

The interactions between Gaze Direction and Group (*p* = .51; individuals with ASD used more averted gaze in both conditions, see Figure 1) and between Phase of Interaction and Group (*p* = .69) were not significant. The interaction between Gaze Direction and Phase of Interaction was significant, *F*(2, 72) = 83.84, *p* < .001 (see Figure 1). In the Gaze At Will condition, GA when ‘thinking’ was significantly greater than when ‘listening’ (*t*(37) = 16.99, *p* < .001; mean listening 23%, thinking 83%) and when ‘speaking’ (*t*(37) = 9.94, *p* < .001; mean speaking 34%). In the Forced Eye Contact condition, GA when ‘thinking’ was also significantly greater than when ‘listening’ (*t*(37) = 5.28, *p* < .001; mean listening 4%, thinking 13%) but the difference between GA during ‘thinking’ and ‘speaking’ did not differ significantly (*p* = .97, mean speaking 13%). The three-way interaction between Gaze Direction × Phase of Interaction × Group did not reach significance (*p* = .27).

Gaze aversion patterns within each phase of the interaction were not significantly correlated with verbal ability (BPVS score), level of functioning (SCQ score) or chronological age in the ASD group (all *p*s > .05). Additionally, there was no significant correlation between patterns of gaze aversion and chronological age or verbal ability in the TD matched group (*p*s > .05).

There was a significant positive correlation between the amount of gaze aversion during the thinking phase and task accuracy in the At Will condition for both the individuals with ASD (*r*(19) = .45, *p* = .054) and the TD matched comparison group (*r*(19) = .52, *p* < .05).^4^ For both groups, increased gaze aversion when thinking was related to increased task accuracy.

#### Williams syndrome

A three-way mixed ANOVA was carried out on the gaze aversion data with factors Group (WS, TD), Gaze Direction (Gaze At Will, Eye Contact), and Phase of Interaction (listening, thinking, speaking). The overall effect of Group was not significant (*p* = .21; mean WS 27%, TD 23%). There was a significant main effect of Gaze Direction, *F*(1, 30) = 83.70, *p* < .001, with more averted gaze in the At Will condition (mean 41%) than the Eye Contact condition (mean 9%). The main effect of Phase of Interaction was also significant, *F*(2, 60) = 104.16, *p* < .001, and post-hoc *t*-tests showed that overall there was significantly less gaze aversion in the listening phase (mean 7%) than the speaking phase (mean 19%; *t*(39) = 3.05, *p* < .01), which in turn showed significantly less gaze aversion than the thinking phase (mean 49%; *t*(39) = 9.12, *p* < .001).

**Figure 2:**
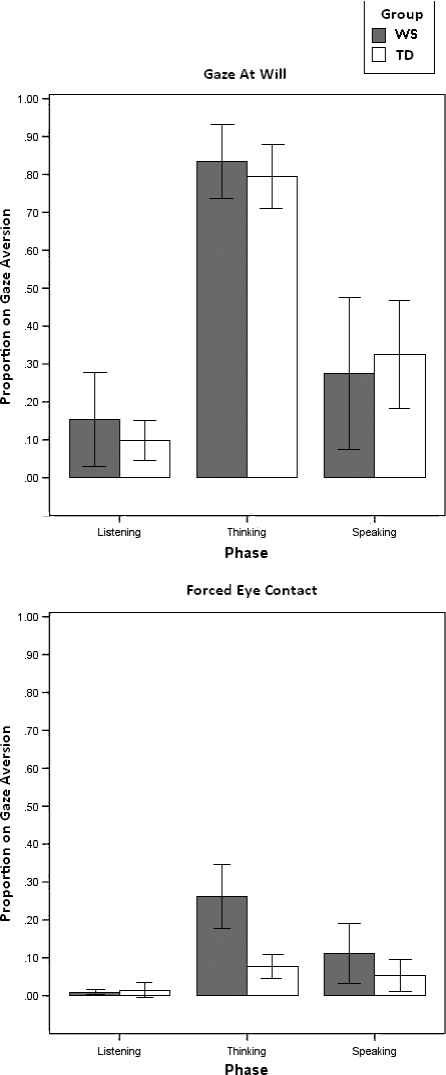
Gaze aversion rates as a function of interaction phase and group for each gaze condition (WS versus TD). Error bars represent standard error of the mean.

The interactions between Gaze Direction and Group (*p* = .38) and Phase of Interaction and Group (*p* = .17) did not reach significance. The interaction between Gaze Direction and Phase of Interaction was significant, *F*(2, 60) = 54.95, *p* < .001; see Figure 2). The three-way interaction between Gaze Direction × Phase of Interaction × Group did not reach significance (*p* = .14).

Gaze aversion patterns within each phase of the interaction were not significantly correlated with verbal ability (BPVS score) or chronological age in the WS group (*p*s > .05) or the TD matches (*p*s > .05).

There was a significant positive correlation between the amount of gaze aversion during the thinking phase and task accuracy in the At Will condition for both the individuals with WS (*r*(16) = .53, *p <* .05) and the TD matched comparison group (*r*(16) = .64, *p* < .01). Increased gaze aversion when thinking was related to increased task accuracy in both groups.

## Discussion

For the first time it has been possible to quantify face interference for individuals with two neuro-developmental disorders that impact upon social attention and cognitive functioning; namely, Williams syndrome (WS) and Autism Spectrum Disorder (ASD). The research allows us to make an important conclusion: Looking at faces interferes with cognitive performance in WS and ASD as well as in typical development. Anyone interacting with an individual who has WS or ASD should expect that the requirement to look at a face will cause a detriment to cognition in the same way that we would expect this in typical development.

The primary outcome measure was task accuracy (and indeed the change in task accuracy) across gaze conditions. The performance levels across groups were similar and were not significantly different. Most importantly, the degree of interference to cognitive processing was statistically comparable across groups. Therefore, we propose that face gaze impacts upon internal cognitive processes in WS, ASD and typical development. However, the neuro-developmental disorder groups found it harder than the typically developing groups to maintain face gaze throughout the interaction. This may imply that if their gaze aversion levels had dropped to ‘typical’ levels, accuracy may have reduced further. Here cognitive capacity may have negatively impacted upon the ability to adhere to the task instruction. Further research is required to make firm conclusions about the equality of interference in typical and atypical development and the current study has emphasized the necessity for such research.

It is important to note that we know from the literature on typical development that greater cognitive interference has been found for looking at faces than other objects (e.g. Doherty-Sneddon *et al*., 2001). However, we do not know if this is the case for individuals who are developing atypically. Therefore, further research is required to unravel whether the current results in the ASD and WS groups are face-specific, related to completion of a secondary dual task *per se*, or indeed are related to the need to switch off from anything in the visual environment. For example, at present there is a lack of existing research to suggest whether the individuals with ASD are struggling more with the basics of saccadic control or the presence of a face within their environment. Research of a very different experimental nature has suggested possible problems with the saccadic control of eye movements in ASD (e.g. Kemner, Verbaten, Cuperus, Camfferman & van Engeland, 1998), but there are no studies of the nature reported here that can be used for direct comparison. The need for further research of this nature is also apparent given that both these atypical populations have deficits of executive functioning, and thus it may be more likely that the face-to-face interference effects we report here are an artefact of dual tasking rather than looking at a face. While this is highly unlikely in the typical control groups we cannot, at present, rule out this interpretation for the individuals with ASD or WS. Most importantly, we propose here that asking individuals with ASD and WS to look at faces while thinking (something they are often asked to do) interferes with concentration, whether this is to do with dual tasking or with faces *per se* is, from a clinical and educational standpoint, less relevant.

It should also be noted that the current study involved the use of mathematics questions and it could be argued that such questions use spatial representations or require the involvement of mental spatial cues. It might be that for this specific type of question eye movements are necessary and indeed helpful to performance. Therefore, an increase in GA when thinking might be related to eye movements involved in the use of mental spatial cues, rather than to avoiding interference from a face. However, existing evidence from typically developing 8-year-olds has shown increases in gaze aversion during phases of thinking for a range of different types of task; including episodic and autobiographical memory (Doherty-Sneddon & Phelps, 2005). It is therefore possible to rule out this suggestion for the typically developing participants in the current study. While there is a lack of existing published data of this nature for individuals who are developing atypically, recent research in our lab has shown increases of gaze aversion while thinking (compared to speaking and listening) when participants with ASD and WS describe a cartoon clip they have watched to an interlocutor, thus involving a more social interaction. We can therefore tentatively suggest that this interpretation of the data is also unlikely for those with ASD and WS in the present study. Further research should manipulate task demands and the nature of the interaction for these atypically developing groups to explore in more detail the impact of question demands on gaze aversion patterns.

Of secondary interest to the current study, and linking to the previously mentioned issues about the control of attention, was the modulation of gaze and adherence to the gaze behaviour instructions. All the participant groups managed to carry out the instruction to hold face gaze: evident by the reduction in GA across conditions for each group. However, as mentioned for the ASD group it was difficult to adhere to the requirement to hold face gaze across all phases of the interaction. Gaze aversion levels remained above those seen in typical development at all interaction stages even though they were lower than those in the gaze ‘At Will’ condition (refer to Figure 1). For individuals with WS, participants managed to hold face gaze (seen by the overall reduction in their GA levels across conditions). However, they found this most difficult during the ‘thinking’ phase of the interaction. They managed this rather well during the listening phase (refer to Figure 2).

Performance during phase 1, when gaze behaviour was not constrained, illustrates that individuals with these neuro-developmental disorders modulate their gaze in accordance with cognitive load in a manner similar to that seen in typical development. Explicitly, more gaze aversion is used when there is a requirement to think about cognitively demanding information than when listening to an interlocutor. This pattern replicates evidence from across the typical developmental spectrum (Doherty-Sneddon *et al*., 2000) as well as recent reports from ASD and WS that involved different individuals (Doherty-Sneddon *et al*., in press). An important insight provided here (and replicating Doherty-Sneddon *et al*., in press) is evidence of atypically increased gaze aversion during the listening phase for individuals with ASD. There are a number of possible interpretations of this finding. For example, it may suggest that individuals with ASD do not recognize the significance of visual perceptual / social communicative cues that can be used to aid speech interpretation. Alternatively, it may be that individuals with ASD need to begin their computations earlier than typically developing individuals due to working memory demands / limitations and thus begin to avert their gaze earlier in the process. Importantly, the results do not suggest that individuals with ASD are ‘aversive’ to holding face gaze and thus the findings of phase 1 have theoretical implications. If aversion to faces was driven by hyper-arousal we would have expected to see increased gaze aversion across all phases of the interaction. However, the individuals with ASD did not avert their gaze all of the time, and there was a distinct modulation of GA between listening, thinking and speaking in the gaze ‘At Will’ condition. Interestingly, there was no significant relationship between level of gaze aversion in the listening phase and SCQ scores for the ASD group, indicating that this did not increase or decrease systematically with severity of ASD. The impact of level of functioning and the relationship with gaze aversion patterns warrants further investigation.

Interestingly, there was a significant correlation between gaze aversion in the thinking phase of the gaze ‘At Will’ condition and task accuracy for individuals with ASD, those with WS and participants who were developing typically. This finding adds to the evidence that gaze aversion is functional in typical development (e.g. see Phelps *et al*., 2006) and provides a new contribution to suggest that this may also be the case for individuals with neuro-developmental disorders such as ASD and WS. When asked this type of moderately difficult mathematics question individuals with ASD, WS and TD showed a benefit of averting their gaze on their task accuracy.

The results of the gaze ‘At Will’ condition also suggest that during person-to-person interactions individuals with WS (here between the ages of 9 and 37 years) are able to modulate their gaze behaviours when appropriate to do so. Therefore, even though individuals with WS may show atypically prolonged attention to faces displayed on computer screens (when the face cannot ‘look back’ and hold mutual gaze; Riby & Hancock, 2008) they are able to modulate their gaze and do not show ‘sticky fixation’ on faces at all points during a real-life interaction.

One possible interpretation of these results across all groups is that the decrease in task accuracy is caused by increased task demands, which may or may not be relevant to the duration of eye contact. Doherty-Sneddon *et al*. (2001) report a series of five studies showing face-to-face interference during communication and memory tasks. Some of these studies included participants being asked to look at the floor, at faces, or at visuo-spatial patterns and all of these conditions could be considered atypical of dual-task. In other studies the same authors included articulatory-suppression as a dual-task condition. The conclusion from this series of studies was that in typical development there were significant and consistent interference effects associated with looking at faces *over and above* any dual-task effects. This was the case even when compared to interference associated with looking at moving visuo-spatial patterns. Interference was evident from both poorer task performance and subjective participant reports. So, typically developing children and adults find that looking at faces makes tasks more difficult than when they are asked to look at something less distracting than a face. The current study was the first to make the suggestion that looking at faces is also distracting for individuals with ASD and WS.

Further research should take forward some of the issues highlighted here to enhance our understanding of the nature, occurrence and consequences of gaze aversion in these neuro-developmental disorders. Importantly, we do not know how information is being processed in the current study and it may be that the *qualitative* nature of the gaze shift is different across the groups. The type of interaction that is studied (e.g. here mathematics questions and answers) may also provide an avenue for investigation. Furthermore, future research would benefit from exploring the impact of level of autistic functioning and intellectual abilities on the strategic use and prevalence of gaze aversion (using a different paradigm from that applied here where it was necessary to constrain recruitment to relatively high functioning individuals due to task demands). Similarly, consideration of individual differences in gaze behaviour and the relationship with cognition are warranted with regard to WS and links to other components of social behaviour. Critically, it is important that the current study be used as groundwork for exploring the developmental trajectory of gaze aversion behaviours in atypical development. Therefore, while these may be limitations of the current work, there is much need for further research within this domain that can contribute to the theoretical, clinical and educational implications of the link between cognition and gaze behaviour in typical and atypical development.

## Footnotes

1 Not counterbalancing the order of conditions was necessary as it was considered that the instruction to maintain face gaze would be difficult to negate and that any immediate gaze aversion in condition 1, if it followed condition 2, may not represent naturally occurring GA levels following such an instruction. Of course participants may have fatigued by condition 2, but it was considered that a total of 12 questions was not of particular demand. In addition any fatigue effects could be alleviated by practice effects.

2 It is worth noting that none of the individuals who participated in this study had previously participated in research reported by Doherty-Sneddon, Riby and Whittle (in press).

3 Note that analysis of the length of the ‘thinking’ phase was also conducted as a function of participant group and gaze condition. This revealed that participants did not change the length of their thinking time as a function of gaze condition. There was also no significant difference between the neuro-developmental disorder groups and their TD matches in terms of the length of the thinking phase. We conducted this analysis to ensure that one group was not ‘giving up’ before the other groups when they were forced to hold face gaze. The analysis suggested that this was not the case.

4 Caution is required for all correlation analyses due to the sample size. Note that it is not appropriate to correlate thinking GA levels and accuracy in the ‘eye contact’ condition as we have controlled / manipulated the participant’s direction of attention.

## Appendix

Example of the types of questions that were asked in the ‘gaze at will’ condition and the comparable question asked in the ‘forced eye contact’ condition. These are example questions that were modified to the specific needs of the individuals and groups (for example, they would be made more simplistic for individuals who had problems with mathematics and numeracy). Importantly, Table 1 of the main manuscript emphasizes that in the ‘gaze at will’ condition, baseline accuracy was comparable across groups and performance was deemed to show that the questions were of moderate difficulty (showing a level of performance similar to that which has shown gaze aversion training benefits in typical development; Phelps *et al*., 2006).

**Table:**

*Condition A*	*Comparable question for Condition B*
What is 86 + 42?	What is 76 + 32?
What is 102 – 24?	What is 112 – 26?
What is 45 + 15?	What is 65 + 25?
What is 33 – 12?	What is 56 – 12?
